# Long-term Success of Cardioneuroablation in a Patient with Tachycardia–bradycardia Syndrome and Syncope

**DOI:** 10.19102/icrm.2021.121001

**Published:** 2021-10-15

**Authors:** Maxim V. Gorev, Shorena G. Nardaia, Olga A. Sergeeva, Elena Yu. Vasilieva, Alexander V. Shpektor, Farkhad G. Rzaev

**Affiliations:** ^1^Cardiology Department, Davydovsky City Hospital, Moscow, Russia; ^2^Cardiology Department, Moscow State University of Medicine and Dentistry, Moscow, Russia

**Keywords:** Atrial fibrillation, atrioventricular block, cardioneuroablation, ganglionated plexus syncope

## Abstract

A case of successful treatment for syncopal episodes caused by intermittent atrioventricular block in a patient with paroxysmal atrial fibrillation/atrial flutter using cardioneuroablation is presented.

## Introduction

Current guidelines are ambiguous with regard to the preferred approach for the treatment of patients with paroxysmal atrial fibrillation (AF) or atrial flutter (AFL) and coincident atrioventricular (AV) conduction impairment. According to the AF treatment guidelines, AF-related bradycardia should be considered as an indication for AF ablation rather than pacemaker (PM) implantation.^1^ On the other hand, the current guidelines for cardiac pacing propose PM implantation if intermittent second- to third-degree AV block causes syncope.^2^

Cardioneuroablation (CNA) is a relatively new method to treat cardioinhibitory neurocardiogenic syncopal and presyncopal episodes. It includes several diagnostics (atropine test, vagal stimulation, and intracardiac mapping) and treatment [endocardial radiofrequency (RF) ablation] “components.”^3^ This method is used to remove the pathological parasympathetic effect on the heart in relatively young patients suffering from cardioinhibitory neurocardiogenic syncopal episodes. Because of the absence of randomized multicenter studies, this approach is not presented in the current guidelines but is used for some patients and in some facilities.^4–6^

We present a case of successful treatment for syncopal episodes caused by an intermittent AV block in a patient with paroxysmal AF/AFL, who was taking a small dose of a non–bradycardia-inducing antiarrhythmic drug.

## Case presentation

### Medical history

**Figure 1:**
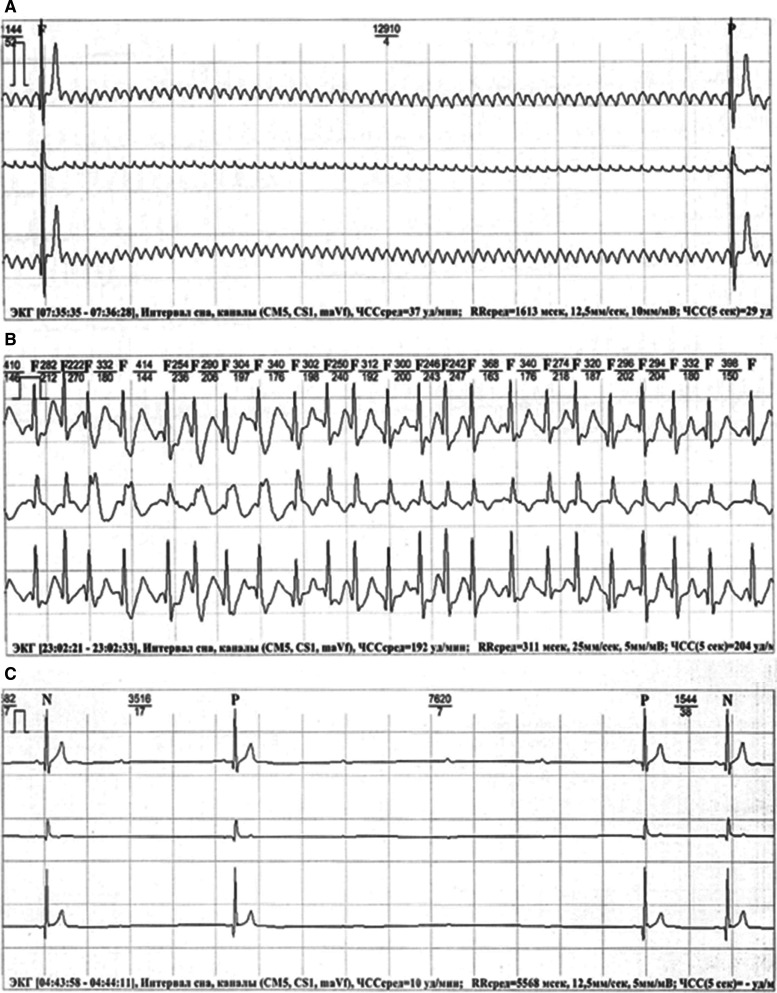
ECG tracings registered during Holter monitoring before ablation. **A:** A pause of 12.9 seconds in duration caused by paroxysmal AV block during AF. **B:** Fast ventricular response during AF, confirming the absence of structural conduction impairment. **C:** AV block developing during night hours simultaneously with SR deceleration most likely resulting from both sinus and AV node suppression by the increased vagal tone.

A 48-year-old male patient with a history of palpitations and syncope was referred to our facility for PM implantation. He had already consulted several other institutions but strongly rejected the proposed PM implantation. He had his first syncopal episode in addition to the first paroxysm of palpitation after participation in an endurance sport (ice hockey training), which resolved spontaneously after several minutes. A similar episode of palpitations occurred a couple of weeks later, lasted several days, and was accompanied by syncope in the first several minutes with subsequent stabilization of hemodynamics and consciousness. Twelve-lead electrocardiography (ECG) and repeat ECG monitoring revealed isthmus-dependent AFL and AF with episodes of a rapid ventricular rate of up to 200 bpm and pauses of up to 13 seconds. Pauses caused by the AV block were also registered in sinus rhythm (SR) **(Figure 1)**.

SR was restored by means of electrical cardioversion, and propafenone was prescribed. Repeat ECG monitoring showed a sinus arrhythmia of 45 to 130 bpm, a wandering atrial PM and intermittent PQ prolongation of up to 420 ms, two sustained AF paroxysms and episodes of 2:1 AV block and third-degree AV block with pauses of up to 7.6 seconds during SR. The patient had strongly rejected the proposed PM implantation. First, we suggested that the patient should agree with the need for permanent pacing, but he again strongly rejected PM implantation. Then, we performed an atropine test to confirm our hypothesis that those pauses and episodes of bradycardia were neurally mediated. This test showed an SR rate acceleration from 58 to 92 bpm and a shortening of the P–Q interval from 160 to 130 ms.

### Pulmonary vein isolation and ganglionated plexus ablation procedure

**Figure 2:**
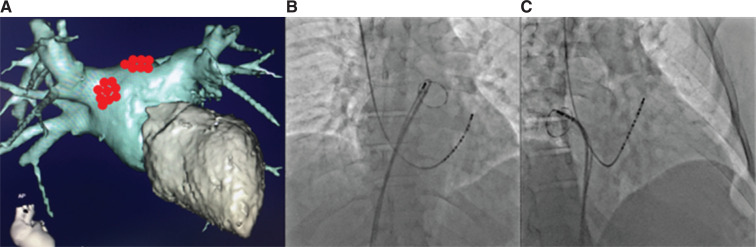
**A:** Three-dimensional reconstruction of LA and left ventricular computed tomography angiography in the anteroposterior view; red dots show the regions of additional RF lesions aiming at the right superior and left superior GPs. **B:** Chest X-ray in the anteroposterior view, demonstrating the position of the mapping catheter during RF ablation in the region of the left superoposterior GP. A Lasso catheter in the left superior PV ostium and a decapolar catheter in the coronary sinus (CS) are also shown. **C:** Chest X-ray in the right oblique view, demonstrating the position of the mapping catheter during RF ablation in the region of the right anterosuperior GP. A Lasso catheter in the right superior PV ostium and a decapolar catheter in the CS are also shown.

These findings led us to the assumption that our experience with SR acceleration during pulmonary vein (PV) isolation (PVI) for AF could be applicable to this patient. We performed an endocardial electrophysiology (EP) study, followed by RF ablation: PVI and cavotricuspid isthmus line. The additional endocardial RF lesions were created anterior to the ostium of the right superior PV (RSPV), where the ganglionated plexus (GP) is located. The left superior GP was ablated on the left atrial (LA) roof near the ostium of the left superior PV (LSPV). Right and left superior GPs were localized by pure anatomical approach under fluoroscopy and a Lasso catheter positioned at the ostia of the RSPV and LSPV, respectively **(Figure 2)**. Fragmented local atrial electrograms were used to confirm the localization of GPs. During these additional lesions, an SR acceleration from 60 to 75 bpm was noted, but at the end of the procedure, the heart rate (HR) decelerated to 68 to 70 bpm. The EP study results prior to and after the ablation are presented in **Table 1**.

### Follow-up

After discharge, the patient was followed up with annually using ECG monitoring and an oral survey on his quality of life, episodes of lightheadedness, and presyncopal and syncopal episodes.

A three-year follow-up off antiarrhythmic therapy revealed neither episodes of palpitations nor syncope. Annual ECG monitoring showed an SR with stable AV conduction without any episodes of bradycardia, pauses, or arrhythmias **(Table 2)**.

## Discussion

The AV block, if not associated with temporary or removable causes, is an indication for PM implantation.^2^ While the long-term positive effect of permanent pacing in patients with symptomatic bradyarrhythmias is widely known, there is a list of early and late complications, which are especially important to consider in young and middle-aged patients with a long life expectancy after the implantation. Usually discussed with patients are the lifelong risks of infections, venous obstruction, lead extraction, AF, and heart failure, which potentially increase the cardiovascular and total mortality levels. But because of the absence of an alternative to PM implantation in the acquired AV block population, these risks are frequently ignored.

The most frequently observed reasons for bradyarrhythmias are HR-decelerating medications (eg, β-blockers, calcium-channel blockers, amiodarone, sotalol), acute myocardial infarction (first 48 hours), inadvertent conduction system injury during transcatheter aortic valve implantation or open heart surgery. In the majority of the remaining cases, the acquired advanced AV block is treated with PM implantation,^2^ although the permanently or intermittently increased parasympathetic tone could also be the reason for sinus node dysfunction or AV conduction disturbances. This factor is not usually screened for in routine clinical practice and frequently stays untreated.

CNA was first described in 2005 by Pachon et al. and is the endocardial RF ablation of regions of GPs in the left and right atria.^3^ It causes the withdrawal of pathologically increased parasympathetic tone from the heart, with subsequent increase of sinus automaticity and acceleration of AV nodal conduction. Young patients with fainting due to the neurocardiogenic syncope with a cardioinhibitory reaction are the most frequent candidates for CNA.^5,7^ This procedure could become an alternative intervention for symptomatic bradyarrhythmia treatment, giving a chance to avoid PM in some patients, but additional investigation and accurate patient selection are necessary prior to the decision about the type of procedure needed.

Middle-aged and older people rarely suffer from neurocardiogenic syncope. But a similar strategy (screening for increased parasympathetic tone) seems equally valuable in tachy–brady patients prescribed for AF ablation. AF guidelines propose ablation in AF-related bradycardia patients^1^ because of the chance of withdrawal of rate-decreasing antiarrhythmic drugs and probable SR acceleration. The latter could be found in some patients and is most likely explained by the incidence of ablation of GPs similar to that performed during CNA.^8–11^ Unfortunately, the incidence of HR acceleration during AF ablation is not absolute. In some patients, pathogenic parasympathetic tone could be eliminated by additional ablation lesions in other regions (superoposterior wall of the right atrium, coronary sinus ostium, right pulmonary artery) guided by the characteristics of local atrial electrograms, fluoroscopy, or non-fluoroscopic three-dimensional mapping.^5^ But none of these lesion sets are included in the regular procedure for AF-related bradycardia.^1^

According to animal experiments, most efferent vagal fibers to the atria travel through the aorta–superior vena cava fat pad, which serves as a “head station” of vagal fibers traveling to both atria.^12^ The right PV fat pad provides vagal innervation of the sinoatrial node, while vagal innervation of the AV node is provided by the inferior vena cava–right atrial fat pad. Similar selective innervation principles were recently demonstrated in humans by Aksu et al.^13^ and Pachon et al.^14^

In our patient, we performed only LA GP ablation. This intraprocedural decision was based on a relatively good response to the ablation of left-sided GPs and the willingness to not expand the procedure volume to the right atrial plexal regions. Retrospectively, we have to admit that this decision was followed by a partial effect (SR acceleration to 75 bpm after CNA and to 92 bpm after atropine), acute (intraprocedural SR deceleration to 65 bpm at the end of the procedure), and delayed (gradual decrease in the mean HR according to annual Holter monitor results) re-innervation. All these effects were previously described by other authors.^15^ But it should also be mentioned that the clinical effect is durable and patients do not complain of arrhythmia recurrence or syncopal episodes.

Finally, this case report contributes to the growing evidence base for the implementation of CNA in clinical practice for the treatment of patients with increased parasympathetic tone, causing symptomatic bradyarrhythmias.

## Conclusions

Patients with intermittent or permanent bradycardia should be additionally investigated, with attention paid to the highest possible HR and the response to atropine infusion. Patients without structural conduction system disease may benefit from non-standard treatment approaches like CNA rather than from PM implantation. Prospective studies evaluating the efficacy risks and benefits of CNA for vagally mediated bradycardia should be considered.

## Figures and Tables

**Table 1:** EP Parameters Prior to and After the CNA

	Before Ablation	After Ablation
Heart rate, bpm	54	71
PQ interval, ms	156	124
AVN ERP, ms	320	280
Antegrade Wenckebach point, ms (bpm)	380 (157)	350 (171)
SNRT	1,480	1,240
cSNRT at 500-ms pacing cycle length*	360	390

AVN ERP: atrioventricular nodal effective refractory period; cSNRT: corrected sinus node recovery time; SNRT: sinus node recovery time.

*cSNRT is corrected by intrinsic cycle length.

**Table 2:** Holter Monitoring Data Before CNA and During Follow-up

	Before CNA			CNA March 13, 2017	One Month Post-CNA (April 11, 2017)	Three Months Post-CNA (June 6, 2017)	One Year Post-CNA (March 13, 2018)	Two Years Post-CNA (July 26, 2019)	Three Years Post-CNA (September 30, 2020)
	August 3, 2016	January 14, 2017	February 20, 2017						
Rhythm	Atrial flutter	SR	SR		SR	SR	SR	SR	SR
Min HR, bpm	21	21	16		59	58	44	43	38
Mean HR, bpm	77	56	52		77	77	65	69	68
Max HR, bpm	204	133	89		111	120	116	126	118
Pause duration, s	12.9	7.6	3.8		No	No	No	No	No

CNA: cardioneuroablation; HR: heart rate; SR: sinus rhythm.

## References

[1] Hindricks G, Potpara T, Dagres N, Arbelo E, Bax JJ, Blomstrom-Lundqvist C (2021). 2020 ESC Guidelines for the diagnosis and management of atrial fibrillation developed in collaboration with the European Association of Cardio-Thoracic Surgery (EACTS): The Task Force for the diagnosis and management of atrial fibrillation of the European Society of Cardiology (ESC) developed with the special contribution of the European Heart Rhythm Association (EHRA) of the ESC. Euro Heart J.

[2] Brignole M, Auricchio A, Baron-Esquivias G (2013). 2013 ESC Guidelines on cardiac pacing and cardiac resynchronization therapy: the Task Force on cardiac pacing and resynchronization therapy of the European Society of Cardiology (ESC). Developed in collaboration with the European Heart Rhythm Association (EHRA). Eur Heart J.

[3] Jose C, Pachon M, Enrique I (2005). “Cardioneuroablation”—new treatment for neurocardiogenic syncope, functional AV block and sinus dysfunction using catheter RF-ablation. Europace.

[4] Debruyne P, Rossenbacker T, Collienne C (2018). Unifocal right-sided ablation treatment for neurally mediated syncope and functional sinus node dysfunction under computed tomographic guidance. Circ Arrhythm Electrophysiol.

[5] Aksu T, Guler TE, Bozyel S, Yalin K (2019). Potential usage of cardioneuroablation in vagally mediated functional atrioventricular block. SAGE Open Med.

[6] Chen YW, Bai R, Lin T (2014). Pacing or ablation: which is better for paroxysmal atrial fibrillation-related tachycardia-bradycardia syndrome?. Pacing Clin Electrophysiol.

[7] Sutton R, Ungar A, Sgobino P (2014). Cardiac pacing in patients with neurally mediated syncope and documented asystole: effectiveness analysis from the Third International Study on Syncope of Uncertain Etiology (ISSUE-3) Registry. Europace.

[8] Sikorska A, Pilichowska–Paszkiet E, Żuk A (2019). Acceleration of sinus rhythm following ablation for atrial fibrillation: a simple parameter predicting ablation efficacy. Kardiol Pol.

[9] Pappone C, Santinelli V, Manguso F (2004). Pulmonary vein denervation enhances long-term benefit after circumferential ablation for paroxysmal atrial fibrillation. Circulation.

[10] Inada K, Yamane T, Tokutake KI (2014). The role of successful catheter ablation in patients with paroxysmal atrial fibrillation and prolonged sinus pauses: outcome during a 5-year follow-up. Europace.

[11] Hocini M, Sanders P, Deisenhofer I (2003). Reverse remodeling of sinus node function after catheter ablation of atrial fibrillation in patients with prolonged sinus pauses. Circulation.

[12] Chiou C-W, Eble JN, Zipes DP (1997). Efferent vagal innervation of the canine atria and sinus and atrioventricular nodes. Circulation.

[13] Aksu T, Guler TE, Bozyel S, Yalin K (2021). Selective vagal innervation principles of ganglionated plexi: step-by-step cardioneuroablation in a patient with vasovagal syncope. J Interv Card Electrophysiol.

[14] Pachon-M EI, Pachon-Mateos JC, Higuti C (2020). Relation of fractionated atrial potentials with the vagal innervation evaluated by extracardiac vagal stimulation during cardioneuroablation. Circ Arrhythmia Electrophysiol.

[15] Pachon-M JC, Pachon-M EI, Pachon CTC (2020). Long-term evaluation of the vagal denervation by cardioneuroablation using holter and heart rate variability. Circ Arrhythmia Electrophysiol.

